# Correction: Budesonide promotes airway epithelial barrier integrity following double-stranded RNA challenge

**DOI:** 10.1371/journal.pone.0306666

**Published:** 2024-07-01

**Authors:** Clara Rimmer, Savas Hetelekides, Sophia I. Eliseeva, Steve N. Georas, Janelle M. Veazey

Fig 5 is uploaded incorrectly. Please see the correct Fig 5 here.

**Fig 5:**
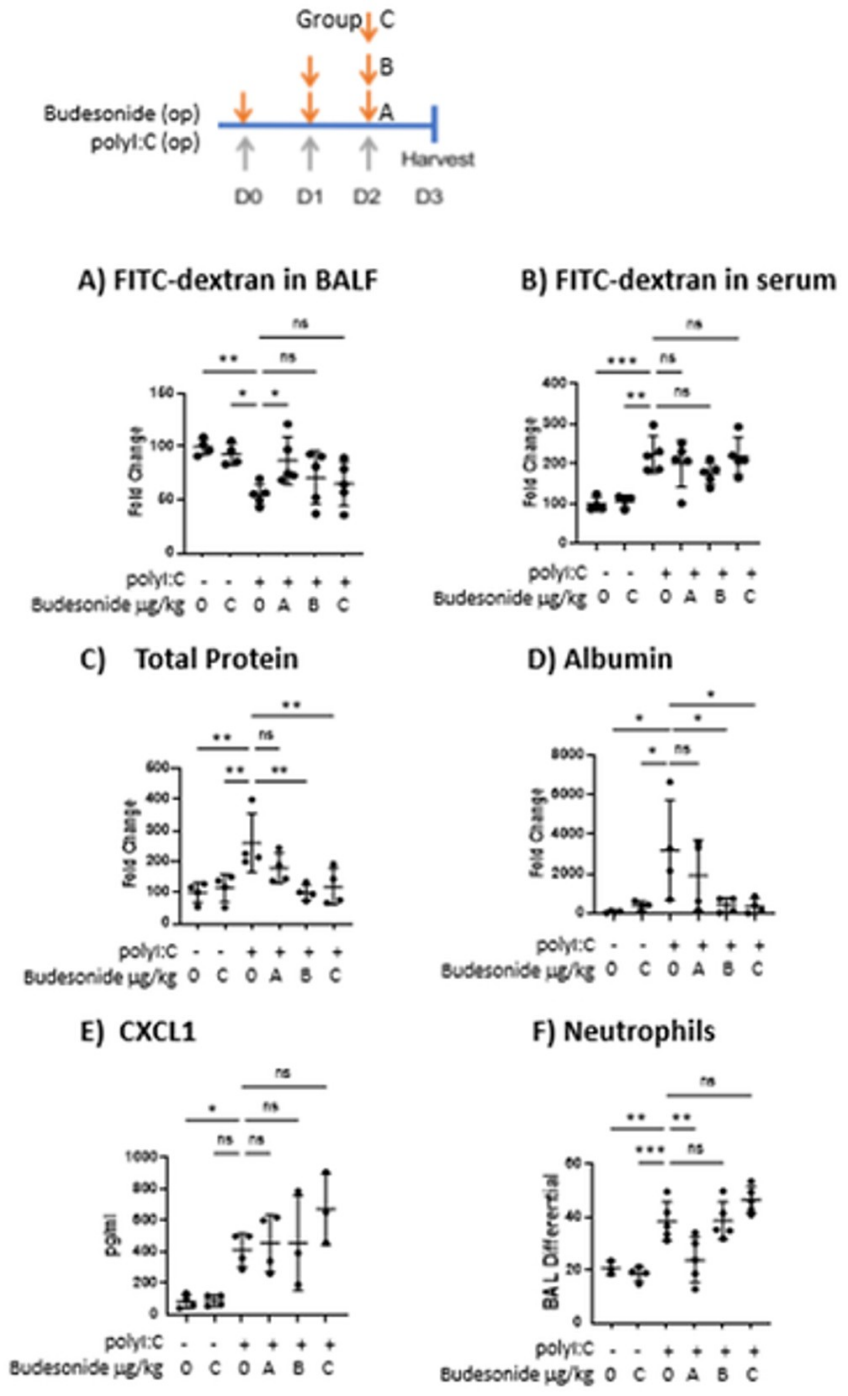
Therapeutic budesonide treatment promotes barrier integrity without hampering neutrophil accumulation. C57B/6 mice were given 10 μg polyI:C o.p. on days 0–2. On either days 0–2 (group A), days 1–2 (group B) or day 2 (group C), mice were also given vehicle or 700 μg/kg budesonide o.p.. On D3, 0.2 mg 4kDa FITC-dextran was instilled o.p. and 1hr later BALF and blood were harvested. A-B) Total protein and albumin levels in BALF were assessed via Bradford and ELISA respectively. C-D) FITC-dextran levels in BALF and serum were analyzed using a fluorescent plate reader. E-F) Neutrophils were assessed via cytospin and H&E staining, and CXCL1 was quantified via ELISA. Data are mean ± standard deviation. One way ANOVA followed by unpaired Tukey’s multiple comparisons test. * p <0.05, **p<0.01, *p<0.001.
