# Supplementary material for: Budesonide promotes airway epithelial barrier integrity following double-stranded RNA challenge
Source: PLoS One. 2021 Dec 6;16(12):e0260706. doi: 10.1371/journal.pone.0260706 (PMC8648122; doi:10.1371/journal.pone.0260706)
Supplement: S1 File — (DOCX) [file pone.0260706.s001.docx]

**Detailed Protocols:**

**16HBE TEER and small molecule flux assay**

1. Seed 1x10^4^ 16HBE cells onto permeable polyester Transwell inserts with 0.4 um pores and 0.33 cm^2^ growth area (Corning; Kennebunk, ME), and culture in DMEM+10% FBS (high glucose Dulbecco’s Minimum Essential Medium with sodium pyruvate and L-glutamine (Life Technologies; Carlsbad, CA), 100 U/ml Penicillin and 100 μg/mL Streptomycin (Life Technologies), and 10 mmol/L HEPES (Life Technologies)) supplemented with 10% fetal bovine serum (Tissue Culture Biologicals; Tulane, CA).
2. Monitor trans-epithelial electrical resistance (TEER) using a voltometer (World Precision Instruments EVOM2) daily until cells reach confluency and TEER is over 800 ohms.
3. Once cells are confluent, aspirate media and apply fresh media containing 1-10 µM budesonide, montelukast, or formoterol.
4. After 18 hr pre-treatment, aspirate media and replace with either fresh media containing either drug alone, or drug plus 0.05 or 0.5 ug/ml high molecular weight polyI:C (InvivoGen Cat#tlrl-pic; Version#11C21-MM).
5. Assess barrier function with TEER at 6, 24, and 48 hrs after polyI:C challenge
6. At 48 hrs after treatment, spike in 4 kDa fluorescein isothiocyanate (FITC) dextran to a final concentration of 10 µg/ml to the apical chamber.
7. At 2 hrs post FITC-dextran addiction, collect an aliquot from the basal chamber to assess the accumulation of FITC-dextran via quantification with a fluorescent plate reader.

**Mice and polyI:C inhalation challenge**

1. Purchase wild-type adult (8-12 weeks old) male and female C57BL/6 were obtained from the National Cancer Institute.
2. Administer budesonide (Sigma CAS51333-22-3) was dosed oropharyngeally (o.p.). at 350 or 700 μg/kg for 1-5 days (depending on the experiment).
   1. Anesthetize mice with isoflurane chamber.
   2. Tie surgical thread to a board and use this to suspend mice by the teeth.
   3. Depress tongue with forceps (to prevent swallowing) and place 50 ul PBS containing 10 ug polyI:C.
   4. Place finger over the nose to encourage the mouse to aspirate the liquid into the lung.
3. Administer 10 μg high molecular weight polyI:C (InvivoGen Cat#tlrl-pic; Version#11C21-MM, oropharyngeally (o.p.) to mice daily for three days to induce robust airway inflammation.
4. Twenty-four hours after the last polyI:C challenge, euthanize mice, collect blood via cardiac puncture and cannulate the trachea for bronchioalveolar lavage fluid (BALF) collection.
   1. BALF was collected by gently pushing 750 ul phosphate buffered saline (PBS) into the lung with a 1 ml syringe attached to a cannula. This PBS flush was performed twice for a total of 1.5 ml collected.
5. Pellet cells recovered in BALF with a 6 min centrifugation at 600xg.
6. Transfer supernatant to a new tube and pellet debris with a 12 min centrifugation at 12,000 rpm at 4^o^C.
7. Resuspend BALF cell pellet in 200 ul PBS and apply 40,000-50,000 cell to cytospin to adhere cells to glass slides.
   1. Stain cells with hematoxylin and eosin (FisherBrand 122-911), allow to dry and count cells.
8. Transfer serum to new tubes and centrifuge for 12 min at 12,000 rpm at 4^o^C.
9. Perform a Bradford Assay on the BALF to quantify total protein according to manufacturer instruction.
10. Perform an ELISA on the BALF to quantify albumin levels (Abcam ab108792 ELISA), and CXCL1 levels (R&D DuoSet DY453 ELISA) according to manufacturer instructions.

**Outside/in leak assay**.

1. At 23 hrs post final polyI:C challenge, and 1 hr prior to harvest, administer 0.2 mg 4kDa FITC-dextran (Sigma#46944) o.p. to mice as described above.
2. Collect BALF as described above.
3. Collect blood via cardiac puncture.
4. Allow blood to coagulate for 10-15 minutes at room temperature before centrifugation for 12 min at 12,000 rpm at 4^o^C to separate serum from cellular component.
5. Transfer 100 ul serum or BALF to an opaque 96 well plate and quantify FITC-dextran with a fluorescent plate reader (Beckman Coulter DTX 880 Multimode plate reader).
