# Supplementary figures and images for: Budesonide promotes airway epithelial barrier integrity following double-stranded RNA challenge
Source: PLoS One. 2021 Dec 6;16(12):e0260706. doi: 10.1371/journal.pone.0260706 (PMC8648122; doi:10.1371/journal.pone.0260706)

## S1. Uncropped western blot images

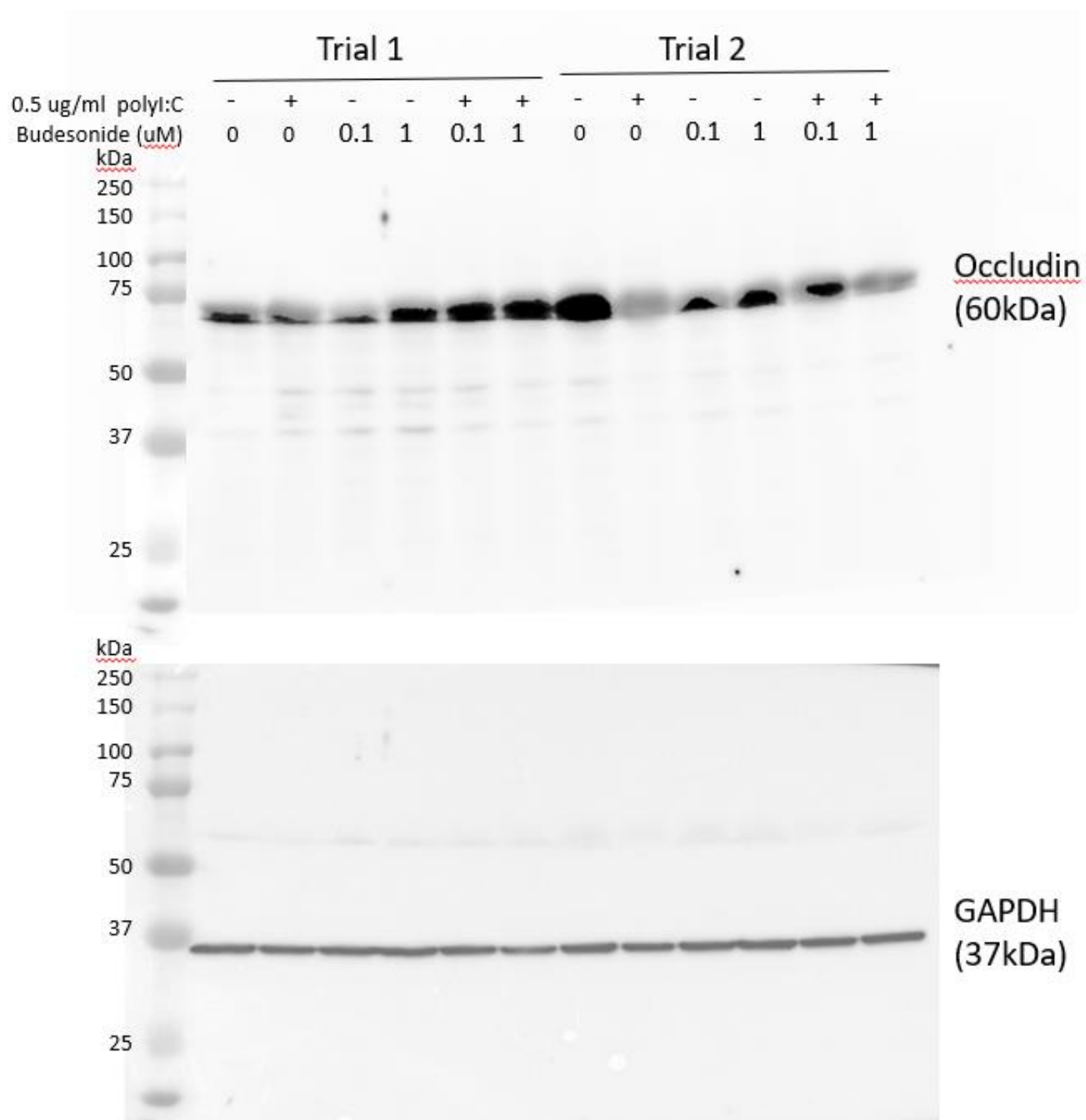

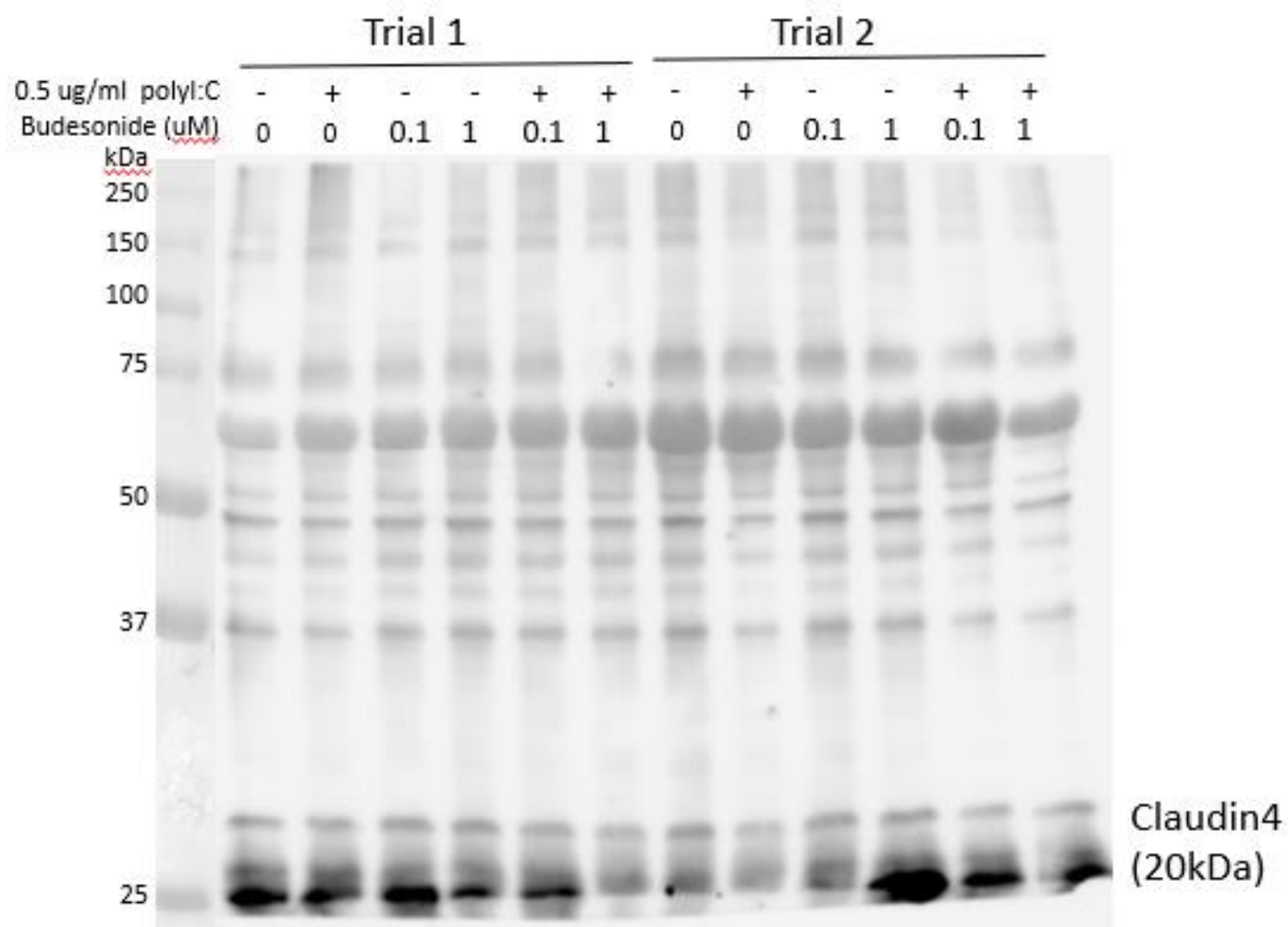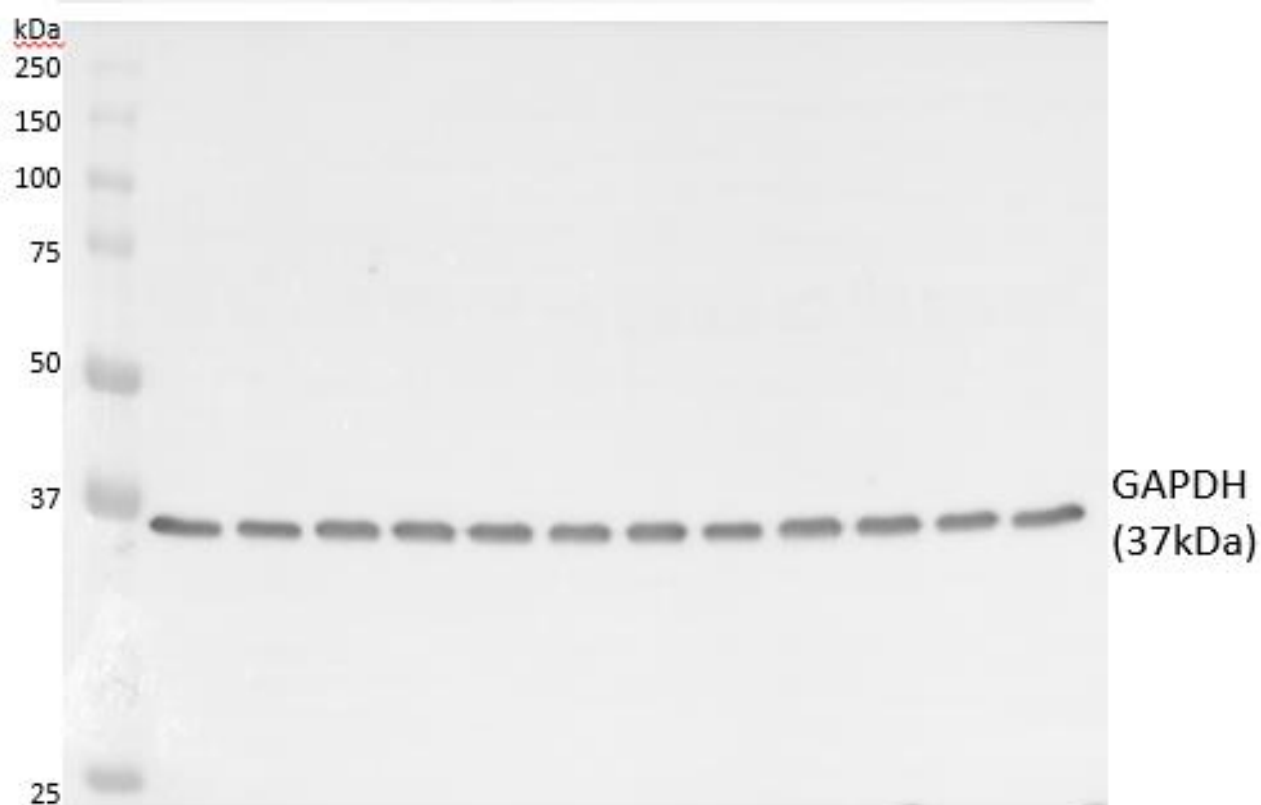

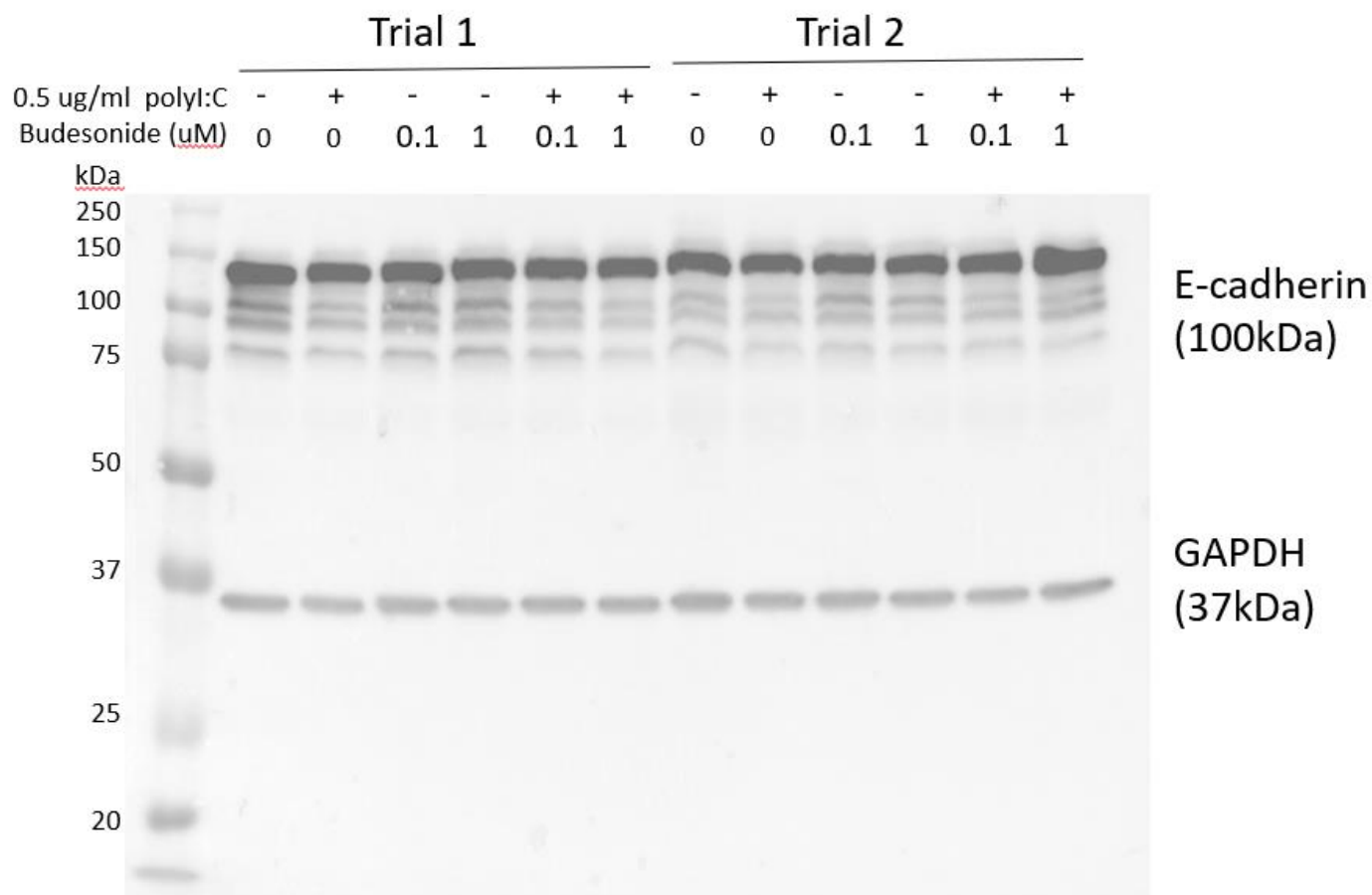

Supplement: S2 File — (PDF) [file pone.0260706.s002.pdf]
