# Supplementary material for: Budesonide promotes airway epithelial barrier integrity following double-stranded RNA challenge
Source: PLoS One. 2021 Dec 6;16(12):e0260706. doi: 10.1371/journal.pone.0260706 (PMC8648122; doi:10.1371/journal.pone.0260706)
Supplement: Attachment — Submitted filename: Response to Reviewers-PLOSONE.docx [file pone.0260706.s004.docx]

PONE-D-21-13593: Response to reviewers
Budesonide promotes airway epithelial barrier integrity following double-stranded RNA challenge

Reviewers' comments:

Reviewer #1:

Comments:
1. The direct effect of budesonide alone on barrier integrity was investigated in vivo; this result is missing from the in vitro studies. On Figure 1 only medium control is shown, but not data on budesonide without virus treatment. These data would be very important for the verification of the barrier integrity inducing effect.

**Response:** Thank you for pointing this out. We analyzed the effects of budesonide alone (0.1 μM) on 16HBE cells in the absence of polyI:C, and did not observe a significant effect on baseline permeability (relative permeability 1.5+/-0.8, n=7 wells, p>0.05).

­2. It would be important to examine the direct effect of budesonide on the barrier forming tight junction proteins. I suggest the authors to complete their work with TJ protein immunocytochemistry.

**Response:** Thank you for this suggestion. In order to study the effects of budesonide on tight junction proteins, we repeated cell culture experiments and analyzed junctional protein expression by Western blot. In revised Figure 2, we now show that budesonide reduced polyIC-induced decreases in the tight junctional proteins occludin and claudin-4. In future experiments, we plan to study tight junction protein expression by immunocytochemistry.

3. More information about airway inflammation and the investigated inflammatory mediators (CXCL1, IL6) should be given in the Introduction.

**Response:** Lines 61-68 have been added (copied here). The host inflammatory response must maintain the delicate balance between sufficient potency to clear infection but avoid excessive inflammation that can lead to barrier disruption and tissue injury [14-16]. Inhaled corticosteroids (ICS) such as budesonide, are commonly prescribed to attenuate airway inflammation and lessen airway hyperreactivity [17-20]. ICS suppress the production of pro-inflammatory cytokines and chemokines in asthma. In asthmatic subjects with neutrophilic airway inflammation, potential targets of ICS include the cytokine interleukin-6 (IL-6) and the neutrophil-attracting chemokine CXCL1. In addition to their role in suppressing airway inflammation, ICS might also promote epithelial barrier integrity, but this has not been as well studied in asthma or models of airway inflammation.

4. At the beginning of the result section there is a sentence referring literature data, please put it to the Introduction.

**Response:** Thank you- this error has been corrected.

5. In case of Fig. 2 the groups do not separate well from each other, this way it is hard to see the treatment groups. To help the readers I suggest another labelling of the TEER curves (e.g. colours).

**Response:** We relabeled the TEER data in Figure 2 with different colors, but the data are still very similar and remain difficult to distinguish. We could re-make this figure as a bar graph if requested by the Reviewer.

6. FITC-dextran permeability is given as % change. Author should calculate and give apparent permeability coefficient (Papp) values in the text (at least).

**Response:** Thank you- this error has been corrected

7. The proper unit of measurement of TEER is Ω × cm2 please give data multiplied with growth surface area of the culture inserts (line 120).

**Response:** Thank you- this error has been corrected.

8. The passage number of the 16HBE cell line should be added to the Material and methods section.

**Response:** Thank you- this error has been corrected.


Minor:
9. Please check and correct the usage of abbreviations:
TEER is abbreviated in line 73, no need to repeat it in line 112
please add abbreviation of bronchoalveolar lavage fluid in line 89
**Response:** Thank you- this error has been corrected.

10. In Material and methods please add the (commercial) source of reagents, plasticware and instruments
give all the details for the Transwell insert type in the main text (line 71)
please add the source of FITC-dextran in line 75
please add the source of the investigated three drugs in lines 77-78)
please specify the type of plate reader in line 76

**Response:** Thank you- these errors have been corrected.

11. The usage of metric units is not consistent:

for “micron” use µ instead of u
for litre: use either l or L but not both

**Response:** Thank you- this error has been corrected.

12. the expression “in vitro” should be written in italic (line 64 and 218)

**Response:** Thank you- this error has been corrected.

13. The word “challenge” is used 19 times in this work. In some of the sentences please use synonyms (e.g. treatment).

**Response:** Thank you again for bringing this to our attention. We have varied the word choice.

Reviewer #2:

1. Paraphrase lines 40-41 in the abstract. As is, makes it sound like tight junctions are adhesion junctions.

**Response:** The wording of lines 40-41 has been changed. It now reads “Airway epithelial cells are normally tightly connected by adherens junctional proteins that join cells together and tight junctional proteins that promote barrier integrity”.

Ion flux has now been replaced with TEER throughout the paper. Thank you.
